# Differential STING and p53 expression in epidermodysplasia verruciformis-associated versus non-EV cutaneous squamous cell carcinomas

**DOI:** 10.1007/s12032-026-03417-0

**Published:** 2026-09-15

**Authors:** Luis Alberto Ribeiro Fróes, Walmar Roncalli Pereira de Oliveira, Paulo Stahlschmidt, Lana Luiza da Cruz Silva, Naiura Vieira Pereira, Mirian Nacagami Sotto

**Affiliations:** 1 Department of Pathology, Faculdade de Medicina da Universidade de São Paulo, São Paulo, Brazil; 2 Department of Dermatology, Faculdade de Medicina da Universidade de São Paulo, São Paulo, Brazil

**Keywords:** Epidermodysplasia verruciformis, Cutaneous squamous cell carcinoma, Betapapillomavirus, STING, p53, BCL2

## Abstract

Epidermodysplasia verruciformis (EV) is a rare genodermatosis characterized by persistent cutaneous β-human papillomavirus infection and increased risk of cutaneous squamous cell carcinoma (cSCC). The cyclic GMP-AMP synthase (cGAS)–stimulator of interferon genes (STING) pathway links cytosolic DNA sensing, genomic instability and innate immune activation, but its expression in EV-associated cSCC remains poorly defined. We quantified STING, p53 and BCL2 immunohistochemical expression in tissue microarrays containing 47 EV-associated and 83 non-EV cSCCs. Tumor H-scores were medians across valid cores; primary comparisons used patient-clustered generalized estimating equations (GEE). STING expression was higher in EV-associated tumors (median H-score 192.4 vs. 149.5); in grade-adjusted GEE, EV status was associated with a + 37.2-unit difference (95% confidence interval [CI] 23.4–51.0; *p* < 0.001). The EV–non-EV STING difference varied by histological grade (interaction *p* = 0.0067), with the largest estimated difference in grade 1 tumors. p53 expression was also higher in EV tumors (97.7 vs. 36.3; adjusted difference + 43.8, 95% CI 18.8–68.8; *p* < 0.001), and EV tumors had higher odds of belonging to a higher p53 tertile (odds ratio [OR] 3.54, 95% CI 1.98–6.32; *p* < 0.001). BCL2 remained low and was not significantly associated with EV (adjusted difference − 1.93, 95% CI − 4.84 to 0.98; *p* = 0.193). STING and p53 remained positively associated after adjustment for histological grade, EV status and patient clustering. These findings define an altered STING protein-expression phenotype in EV-associated cSCC, accompanied by increased p53 expression but no independent BCL2 association, and support functional investigation of cGAS–STING signaling.

**Supplementary Information:**

The online version contains supplementary material available at https://doi.org/10.1007/s12032-026-03417-0.

## Introduction

Epidermodysplasia verruciformis (EV) is a rare inherited disorder characterized by lifelong susceptibility to cutaneous β-human papillomavirus (β-HPV) infection, disseminated flat wart-like lesions and increased risk of cutaneous squamous cell carcinoma (cSCC), particularly on sun-exposed skin. Genetic studies have linked EV to loss-of-function variants in TMC6/TMC8 and to disruption of the CIB1–EVER1–EVER2 complex, supporting a model in which defective keratinocyte-intrinsic immunity permits persistent β-HPV infection and virus-associated carcinogenesis [1, 2].

The cGAS–STING pathway is relevant to this setting because it links cytosolic DNA sensing, innate immune activation and cellular responses to genomic stress. cGAS detects cytosolic double-stranded DNA and activates STING-dependent type I interferon signaling [3]. Cytosolic DNA may also arise from micronuclei generated by chromosomal instability, thereby connecting DNA damage to innate immune activation [4]. In HPV-positive keratinocyte models maintaining viral episomes, cGAS–STING signaling remains responsive to cytosolic DNA, and cGAS participates in DNA-damage-induced apoptosis, supporting a functional intersection between HPV persistence, innate DNA sensing and genotoxic stress [5]. Despite this biological rationale, quantitative STING protein expression in EV-associated cSCC relative to non-EV cSCC remains poorly characterized.

p53 provides complementary context for the DNA-damage response in EV-associated cSCC. TP53 is one of the most frequently mutated driver genes in primary cSCC [6]. Abnormal p53 expression together with TP53 mutations has also been documented specifically in EV-associated viral skin tumors [7]. Previous immunohistochemical work evaluated p53 and BCL2 across a broader spectrum of keratinocytic hyperproliferative lesions that included three non-tumorigenic EV lesions, but did not specifically compare EV-associated cSCC with non-EV cSCC [8]. BCL2 was assessed as an additional apoptosis-related marker; in this context, β-HPV E6 proteins, including those from HPV-5 and HPV-8, β-HPV types classically associated with EV, can degrade the pro-apoptotic BCL2-family member Bak and protect keratinocytes from UVB-induced apoptosis [9]. BCL2 expression has also been characterized immunohistochemically in non-melanoma skin cancer [10].

Accordingly, this study aimed to compare the immunohistochemical expression of STING, p53 and BCL2 between EV-associated and non-EV cSCC. We quantified these markers by digital immunohistochemistry in tissue microarrays of both groups, assessing between-group differences, histological grade-related patterns and marker correlations.

## Materials and methods

### Sample selection

We retrospectively analysed archival cutaneous squamous cell carcinoma (cSCC) specimens from the Laboratory of Dermatopathology, Department of Dermatology, Faculdade de Medicina da Universidade de São Paulo, São Paulo, Brazil. The cohort comprised 47 EV-associated cSCCs and 83 non-EV, non-immunocompromised cSCCs. EV was diagnosed on the basis of a characteristic clinical history of disseminated flat wart-like lesions beginning in childhood together with compatible histopathological findings. Patient linkage was available for 46 of the 47 EV tumors, corresponding to 13 patients; some EV patients contributed multiple tumors, whereas each NEV tumor originated from a different patient. Patient-clustered and patient-level analyses were restricted to the 46 linked EV tumors. Previous molecular investigation had confirmed β-HPV infection in 9 of the 13 EV patients, who contributed 37 of these 46 tumors [11]. Tissue microarrays (TMAs) were constructed from representative tumor areas, with up to four cores available per tumor. All cases were independently reviewed by two dermatopathologists.

### Immunohistochemistry

Immunohistochemistry was performed on formalin-fixed, paraffin-embedded TMA sections using anti-TMEM173/STING (Abcam, Cambridge, UK; ab92605), anti-p53 clone Bp53-11 (Roche Tissue Diagnostics; 760–2542), and anti-BCL2 clone 124 (Roche Tissue Diagnostics; 790–4464). STING was detected using the Novolink Max Polymer Detection System, whereas p53 and BCL2 were stained on the Ventana automated IHC/ISH platform. For each marker, EV and NEV samples were immunostained within the same staining batch under identical experimental conditions. Appropriate positive and negative controls were included; additional staining details are provided in the **Supplementary Methods.**

### Digital image acquisition and quantification

Immunostained TMA slides were digitized using the Aperio ScanScope CS system (Vista, CA, USA) and analyzed in QuPath v0.6.0 [12]. TMA cores were identified with the dearrayer tool and manually checked for tissue adequacy and artifacts. Image analysis was performed blinded to EV/NEV group allocation and restricted to manually delineated tumor areas. Marker-specific scoring used nuclear DAB optical density for p53 and cytoplasmic DAB optical density for STING and BCL2. Detected tumor cells were classified as negative, 1+, 2+, or 3 + using fixed DAB optical-density thresholds applied uniformly across slides; threshold values and calibration procedures are provided in the **Supplementary Methods**. H-scores were calculated per core as 1 × (% weak) + 2 × (% moderate) + 3 × (% strong), yielding values from 0 to 300. Cores with fewer than 100 detected tumor cells were excluded, and the tumor-level H-score for each marker was defined as the median across valid cores.

### Statistical analysis

The tumor was the primary observational unit. Because some EV patients contributed multiple tumors, primary inferential comparisons of tumor-level H-scores used generalized estimating equation (GEE) models with patient identity as the clustering variable to account for within-patient correlation. EV–NEV differences in continuous H-scores were evaluated in unadjusted and histological grade-adjusted models. Multivariable models additionally adjusted for age at biopsy, sex, histological grade, and anatomical region. Effect modification by histological grade was assessed using EV × grade interaction terms. Associations of EV status with STING and p53 tertiles were evaluated using cluster-aware ordinal logistic regression, and the association between STING and p53 H-scores was assessed using patient-clustered GEE models. Sensitivity analyses included patient-level aggregation, restriction to tumors with at least two valid cores, age-overlap analyses, alternative across-core aggregation methods, and the original unclustered tumor-level comparisons; full specifications are provided in the **Supplementary Methods.** Benjamini–Hochberg false discovery rate correction was applied within predefined families of tests, with statistical significance set at *p* < 0.05.

## Results

The archival cohort comprised 47 EV-associated and 83 non-EV cSCCs. Patient-clustered analyses included 46 EV tumors from 13 patients and all 83 NEV tumors. EV tumors occurred at younger ages (median 54.0 [IQR 43.0–62.0] vs. 78.0 [69.0–83.5] years); other clinicopathological characteristics are summarized in **Supplementary Table 1.**

**Fig. 1:**
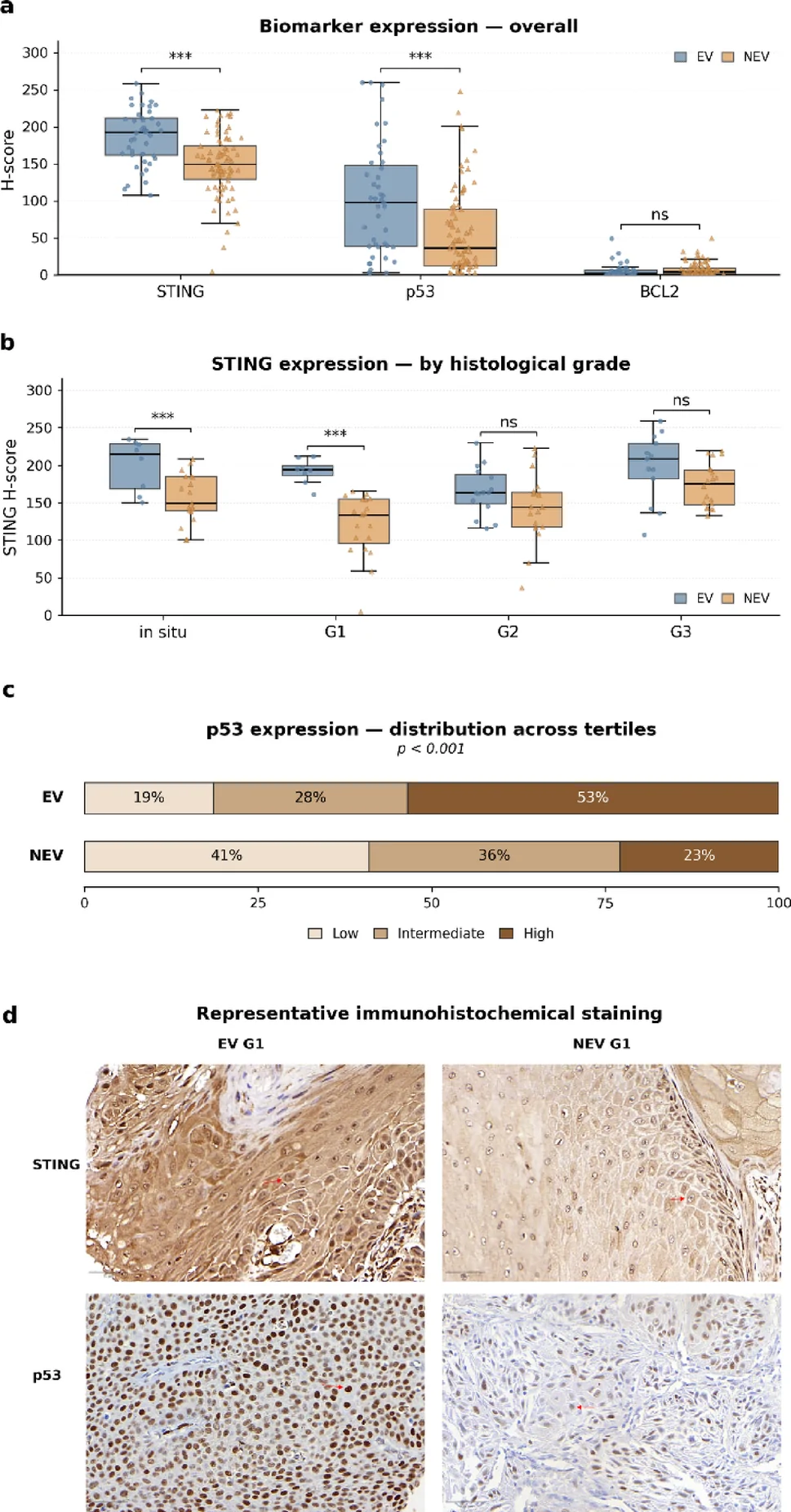
Quantitative biomarker profile in EV-associated and non-EV cutaneous squamous cell carcinomas. (**a**) Tumor-level STING, p53 and BCL2 H-scores; annotations are from grade-adjusted patient-clustered GEE models. (**b**) STING H-scores by histological grade; annotations are grade-specific GEE contrasts, with a significant global EV × grade interaction (*p* = 0.0067). (**c**) Global p53 tertile distribution; *p* < 0.001 from patient-clustered ordinal logistic regression. (**d**) Representative 20× STING and p53 immunohistochemistry in G1 EV and NEV tumors; red arrows indicate cytoplasmic STING or nuclear p53 immunoreactivity; scale bars, 50 μm. H-scores are per-tumor medians across valid TMA cores. EV, epidermodysplasia verruciformis-associated; NEV, non-EV; TMA, tissue microarray. ***p < 0.001; ns, not significant

STING expression was higher in EV than non-EV tumors (median H-score 192.4 [IQR 161.9–211.8], *n* = 44 vs. 149.5 [129.2–173.9], *n* = 78). In grade-adjusted patient-clustered GEE, the EV–non-EV difference was + 37.2 H-score units (95% CI 23.4–51.0; *p* < 0.001). p53 was likewise higher (97.7 [38.4–147.8], *n* = 43 vs. 36.3 [12.7–89.6], *n* = 83; adjusted difference + 43.8, 95% CI 18.8–68.8; *p* < 0.001). BCL2 remained low and did not differ significantly (2.47 [0.74–7.06], *n* = 44 vs. 4.44 [1.61–9.90], *n* = 82; adjusted difference − 1.93, 95% CI − 4.84–0.98; *p* = 0.193) **(**Table 1; Fig. 1a**).**

**Table 1:** Tumor-level biomarker expression and patient-clustered GEE analyses in EV-associated and non-EV cSCC

Marker	EV median [IQR] (*n*)	NEV median [IQR] (*n*)	Unadjusted GEE difference (95% CI); *p*	Grade-adjusted GEE difference (95% CI); *p*
STING	192.4 [161.9–211.8] (44)	149.5 [129.2–173.9] (78)	+ 37.56 (23.67–51.46); *p* < 0.001	+ 37.20 (23.44–50.97); *p* < 0.001
p53	97.7 [38.4–147.8] (43)	36.3 [12.7–89.6] (83)	+ 44.01 (17.72–70.30); *p* = 0.001	+ 43.78 (18.76–68.80); *p* < 0.001
BCL2	2.47 [0.74–7.06] (44)	4.44 [1.61–9.90] (82)	−2.06 (− 4.91–0.78); *p* = 0.155	−1.93 (− 4.84–0.98); *p* = 0.193

Values are tumor-level median H-score [IQR] (number of evaluable tumors) in the patient-linked analytic dataset. GEE models were fitted at the tumor level with patient identity as the clustering variable and robust standard errors; grade-adjusted models additionally included histological grade. CI, confidence interval; cSCC, cutaneous squamous cell carcinoma; EV, epidermodysplasia verruciformis-associated; GEE, generalized estimating equation; IQR, interquartile range

The EV–non-EV STING difference varied by histological grade (EV × grade Wald χ²(3) = 12.20, *p* = 0.0067) (Fig. 1b). Model-estimated differences were + 43.8 H-score units in situ, + 72.2 in G1, + 22.2 in G2 and + 23.8 in G3; the G1 effect exceeded those in G2 and G3 after Holm correction (*p* = 0.0138 and *p* = 0.0366). No significant EV × grade interaction was observed for p53 (*p* = 0.162; **Supplementary Table 2**).

The p53 tertile distribution also differed by EV status (Fig. 1c), with EV tumors enriched in the high tertile (53.5% vs. 22.9%) and depleted in the low tertile (18.6% vs. 41.0%). Cluster-aware ordinal regression showed higher odds of a higher p53 tertile in EV tumors (OR 3.54, 95% CI 1.98–6.32; *p* < 0.001), persisting after grade adjustment (**Supplementary Table 3**). STING and p53 H-scores were positively correlated (Spearman ρ = 0.44), and the association persisted after adjustment for grade, EV status and patient clustering (*p* = 0.00145).

In multivariable patient-clustered models adjusted for age, sex, histological grade and anatomical region, EV status remained associated with higher STING (+ 49.4 H-score units, 95% CI 27.5–71.3; *p* < 0.001) and p53 (+ 48.8, 95% CI 23.8–73.7; *p* < 0.001), whereas BCL2 remained non-significant. Findings were concordant in patient-level aggregation, age-overlap, ≥ 2-valid-core and alternative across-core aggregation sensitivity analyses (**Supplementary Tables 4 and 5**).

## Discussion

This study evaluated STING, p53 and BCL2 immunohistochemical expression in epidermodysplasia verruciformis (EV)-associated cutaneous squamous cell carcinomas (cSCC) compared with non-EV cSCC controls (NEV), using digital H-score quantification and per-tumor aggregation across valid TMA cores. STING was the principal finding, with higher expression in EV-associated tumors and a significant EV × histological-grade interaction. p53 expression was also higher in EV tumors, whereas BCL2 showed no independent association with EV. STING and p53 remained positively associated after adjustment for histological grade, EV status and patient clustering.

### STING expression in EV-associated cSCC

Higher STING expression was the most consistent difference between EV and NEV tumors. Importantly, the EV–NEV effect varied by histological grade in the formal interaction model (*p* = 0.0067), with the largest estimated difference in G1 tumors; this effect was significantly greater than those in G2 and G3. A substantial EV–NEV difference was also observed in situ. These cross-sectional findings indicate heterogeneity of the EV–NEV STING association across histological categories.

The cGAS–STING pathway is a central innate immune sensor of cytosolic DNA: cGAS detects double-stranded DNA and generates cGAMP, which activates STING and downstream TBK1–IRF3 signaling [3, 13]. Cytosolic DNA may derive from viral infection or from host genomic instability, including micronuclei generated by aberrant mitoses [4]. Both triggers are biologically relevant in EV, a condition characterized by abnormal susceptibility to chronic infection by oncogenic β-genus papillomaviruses and by interaction between β-HPV oncoproteins and ultraviolet-induced DNA damage. These mechanisms provide a biological rationale for altered STING abundance in EV-associated cSCC, but the present cross-sectional data do not determine whether this phenotype reflects a constitutive feature of EV skin or a change acquired during malignant transformation.

Total STING immunohistochemistry measures protein abundance rather than functional pathway activation. Brooke et al. [14] demonstrated that β-HPV-8 E6 reduces STING phosphorylation and attenuates downstream innate immune signaling in keratinocytes exposed to cytosolic DNA, despite cGAS being recruited to micronuclei. Thus, our findings define an altered STING protein-expression phenotype, and increased STING abundance does not imply intact downstream signaling. In a large immunohistochemical survey, Menz et al. [15] documented heterogeneous STING expression in cutaneous cSCC; our study extends this literature by quantitatively comparing EV-associated and non-EV tumors.

### p53 expression in EV-associated cSCC

p53 expression was also higher in EV tumors, with enrichment in the upper tertile. No significant EV × histological-grade interaction was detected. However, p53 immunohistochemistry cannot distinguish wild-type p53 stabilized by cellular stress from accumulation of mutant p53. Although TP53 alterations are common in cSCC [16], the present staining data cannot establish TP53 mutational status, and archival material was not available for sequencing. Thus, the p53 findings define a protein-expression phenotype rather than TP53 mutational status.

### STING–p53 correlation: convergent stress signaling

STING and p53 remained positively associated after adjustment for histological grade, EV status and patient clustering. This association does not establish direct co-regulation but is consistent with experimental evidence linking p53 status to STING signaling: p53 activation can increase STING expression [17], whereas mutant p53 can disrupt STING–TBK1–IRF3 signaling [18]. The observed association is also compatible with shared DNA-damage-related or senescence-associated stress [19].

### BCL2 expression

BCL2 expression was very low in both groups and showed no independent association with EV in patient-clustered GEE models. This is consistent with prior immunohistochemical evidence of low BCL2 expression in cSCC [10].

### Translational implications and future directions

The present findings do not support treatment selection based on STING or p53 immunohistochemistry alone. Future studies should prioritize functional assessment of the cGAS–STING axis using phospho-STING, phospho-TBK1, phospho-IRF3 and interferon-stimulated gene readouts, ideally across established cSCC, non-neoplastic EV skin and precursor lesions. Comprehensive β-HPV typing and viral-load assessment, together with TP53 sequencing, would clarify the viral and molecular context of the observed phenotype. Pharmacological modulation of cGAS–STING should be investigated only after functional differences are established.

### Limitations

This retrospective, single-institution study included repeated tumors from several EV patients; clustering and patient-level sensitivity analyses were therefore used, but the number of unique EV patients remained limited. EV tumors also arose at substantially younger ages than NEV tumors; associations persisted after multivariable adjustment and age-overlap analysis, although residual confounding remains possible. Individual cumulative UV exposure was unavailable. Historical β-HPV confirmation covered only part of the EV cohort, without tumor-level typing or viral-load data, and HPV was not molecularly assessed in NEV tumors. Total STING staining does not establish pathway activation, p53 staining does not define TP53 status, and the cross-sectional design precludes temporal inference. Some histological-grade strata were also small.

Overall, EV-associated cSCCs show a distinct protein-expression profile dominated by increased STING, with accompanying p53 elevation and no independent BCL2 association with EV. Functional and molecular studies are needed to determine the mechanisms underlying this phenotype.

## Supplementary Information

Below is the link to the electronic supplementary material.

Supplementary Material 1

Supplementary Material 2

## Data Availability

The data supporting the findings of this study are provided as Supplementary Table 1, containing per-tumor median H-scores for STING, p53 and BCL2 in all 130 tumors. Additional data are available from the corresponding author on reasonable request, subject to institutional and ethical restrictions.
