# Supplementary material for: Differential STING and p53 expression in epidermodysplasia verruciformis-associated versus non-EV cutaneous squamous cell carcinomas
Source: Med Oncol. 2026 Sep 15;43(10):286. doi: 10.1007/s12032-026-03417-0 (PMC13578048; doi:10.1007/s12032-026-03417-0)
Supplement: Supplementary file 1 [file 12032_2026_3417_MOESM2_ESM.pdf]

Online Resource 1

Supplementary Methods and Results

Article title: Differential STING and p53 expression in epidermodysplasia verruciformis-associated versus non-EV cutaneous squamous cell carcinomas

Authors: Luis Alberto Ribeiro Fróes Jr.; Walmar Roncalli Pereira de Oliveira; Paulo Stahlschmidt; Lana Luiza da Cruz Silva; Naiura Vieira Pereira; Mirian Nacagami Sotto

Corresponding author: Luis Alberto Ribeiro Fróes Jr.

Email: lulafroes@usp.br

Supplementary Methods

Cohort linkage and repeated tumors. The original archival cohort included 47 epidermodysplasia verruciformis (EV)-associated cutaneous squamous cell carcinomas (cSCCs) and 83 non-EV cSCCs. Patient linkage was available for 46 of the 47 EV tumors, corresponding to 13 patients. Patient-clustered and patient-level analyses were based on these 46 linked EV tumors. Multiple tumors were available from six EV patients, whereas each of the 83 non-EV tumors originated from a different patient. The numbers of tumors contributed by the 13 linked EV patients were 22, 6, 3, 3, 3, 2, 1, 1, 1, 1, 1, 1, and 1. Historical molecular investigation had confirmed β-HPV infection in 9 of the 13 linked EV patients, who contributed 37 of the 46 linked tumors. HPV status was not molecularly assessed in the non-EV group.

Immunohistochemistry and digital image analysis. Formalin-fixed, paraffin-embedded tissue specimens had been fixed in 10% neutral-buffered formalin (pH 7.4). Immunohistochemistry was performed on tissue microarray (TMA) sections using anti-TMEM173/STING (Abcam, Cambridge, UK; ab92605), anti-p53 clone Bp53-11 (Roche Tissue Diagnostics; 760-2542), and anti-BCL2 clone 124 (Roche Tissue Diagnostics; 790-4464). STING was detected using the Novolink Max Polymer Detection System (Leica Biosystems, Newcastle upon Tyne, UK; K0690), whereas p53 and BCL2 were stained on the Ventana automated IHC/ISH platform. Antigen retrieval, chromogen development, and counterstaining followed institutional staining protocols. For each marker, EV and non-EV samples were immunostained within the same staining batch under identical experimental conditions. Positive controls comprised breast carcinoma for STING and lymph node tissue for p53 and BCL2. Negative controls were performed by omission of the primary antibody and substitution with non-reactive isotype-matched serum.

Immunostained TMA slides were digitized using the Aperio ScanScope CS system (Vista, CA, USA) and analysed in QuPath v0.6.0. TMA cores were identified using the dearrayer tool and manually checked for grid accuracy, tissue adequacy, and artifacts. Image analysis was performed blinded to EV/non-EV group allocation. Tumor areas were manually delineated within each evaluable core, and cell detection was restricted to these tumor annotations using QuPath's built-in cell detection algorithm and color deconvolution to separate hematoxylin and 3,3′-diaminobenzidine (DAB) signals. Nuclear DAB optical density was used for p53 and cytoplasmic DAB optical density for STING and BCL2.

Detected tumor cells were classified using fixed DAB optical-density thresholds applied uniformly across slides: negative, <0.053; weak (1+), 0.053 to <0.112; moderate (2+), 0.112 to <0.221; and strong (3+), ≥0.221. Thresholds were calibrated visually against pathologist-defined reference regions spanning negative, weak, moderate, and strong staining and were then fixed for the complete analysis. H-scores were calculated per

core as 1 × (% weak) + 2 × (% moderate) + 3 × (% strong), yielding values from 0 to 300. Cores with fewer than 100 detected tumor cells within the tumor annotation were excluded from scoring. For each tumor and marker, the primary tumor-level H-score was the median across valid cores. Arithmetic-mean and detection-weighted-mean H-scores across valid cores were evaluated as alternative across-core aggregation methods; the detection-weighted mean weighted each core by its number of detected tumor cells. Core evaluability and exclusions by marker and study group are summarized in Supplementary Table 5.

Statistical analysis. The tumor was the primary observational unit. Analyses requiring patient identity were restricted to the 46 linked EV tumors and the 83 non-EV tumors, each of which originated from a distinct patient. Primary comparisons of continuous H-scores between EV and non-EV tumors used Gaussian identity-link generalized estimating equation (GEE) models with patient identity as the clustering variable, a working-independence correlation structure, and robust standard errors. Models were fitted first with EV status as the exposure of interest and subsequently with adjustment for histological grade as a four-level categorical covariate.

Multivariable patient-clustered GEE models additionally adjusted for age at biopsy, sex, histological grade, and anatomical region, categorized as head/neck, trunk, or extremities. A sensitivity analysis restricted the cohort to the overlapping age range of 46–70 years.

Effect modification by histological grade was evaluated using EV × grade interaction terms. For STING, the interaction model used patient-clustered GEE with working independence and robust standard errors. Holm adjustment was applied to the six pairwise comparisons of grade-specific EV–non-EV effects when the global interaction was significant. For p53, the primary interaction test used an exchangeable working correlation structure; working-independence and bias-reduced covariance analyses were used as sensitivity analyses.

Associations between STING and p53 H-scores were evaluated using patient-clustered Gaussian identity-link GEE models, first with adjustment for histological grade and subsequently with adjustment for histological grade and EV status.

For categorical analyses, global tertiles were defined separately for STING and p53 from the pooled evaluable tumor distributions in the patient-linked dataset. Ordered tertile membership was analysed using ordinal logistic regression with standard errors clustered by patient, both without and with adjustment for histological grade. A separate exploratory p53 classification used H-score categories of <15, 15–150, and >150.

Sensitivity analyses included patient-level aggregation, unclustered tumor-level comparisons, restriction to tumors represented by at least two valid cores, and alternative across-core aggregation methods. For patient-level aggregation, tumor-level H-scores were reduced to a single median value per patient for each biomarker and EV–non-EV differences were evaluated using Mann–Whitney U tests with Cliff's δ and bootstrap 95% confidence intervals based on 10,000 resamples; Benjamini–Hochberg false discovery rate (FDR) correction was applied across the three marker comparisons. Unclustered tumor-level sensitivity analyses used Mann–Whitney U tests with Cliff's δ and bootstrap 95% confidence intervals, with grade-adjusted comparisons performed using van Elteren tests stratified by histological grade. Benjamini–Hochberg FDR correction was applied within predefined families of related analyses where indicated. Statistical significance was defined as a two-sided $p < 0.05$. Analyses were performed using Python 3.11 with the SciPy and StatsModels libraries.

Supplementary Results

Patient-clustered and patient-level analyses. In tumor-level GEE models clustered by patient, EV status remained associated with higher STING and p53 H-scores. Unadjusted EV–non-EV differences were +37.56 H-score units for STING (95% CI 23.67–51.46; $p=1.17\times10^{-7}$) and +44.01 for p53 (17.72–70.30; p=0.00103); BCL2 was not significant (−2.06, −4.91–0.78; p=0.155). Grade-adjusted estimates were similar: +37.20 for STING (23.44–50.97; $p=1.17\times10^{-7}$), +43.78 for p53 (18.76–68.80; p=0.00061), and −1.93 for BCL2 (−4.84–0.98; p=0.193) (Supplementary Table 4). Patient-level median aggregation was concordant: STING remained higher in EV patients (195.45 vs 149.49; FDR-adjusted p<0.001; Cliff's δ=0.66), as did p53 (97.09 vs 36.25; FDR-adjusted p=0.0049; δ=0.55), whereas BCL2 showed no meaningful separation (5.48 vs 4.44; FDR-adjusted p=0.892; δ=−0.03).

Multivariable and age-overlap analyses. After adjustment for age at biopsy, sex, histological grade and anatomical region, EV status remained associated with higher STING (+49.4 H-score units, 95% CI 27.5–71.3; $p=9.7\times10^{-6}$) and p53 (+48.8, 23.8–73.7; $p=1.27\times10^{-4}$), while BCL2 remained non-significant (+0.04, −4.3–4.4; p=0.987) (Supplementary Table 4). Restriction to the overlapping age range of 46–70 years yielded the same pattern: STING $p=2.8\times10^{-7}$, p53 $p=1.17\times10^{-9}$, and BCL2 p=0.77 (Supplementary Table 5).

Histological-grade analyses. The global STING EV × grade interaction was significant (Wald $\chi^2(3)=12.20$; p=0.0067). Model-estimated EV–non-EV differences were +43.8 H-score units in situ (95% CI 21.4–66.2), +72.2 in G1 (51.0–93.5), +22.2 in G2 (−4.7–49.2), and +23.8 in G3 (−1.1–48.6). Holm-adjusted comparisons showed a larger EV–non-EV effect in G1 than G2 (p=0.0138) or G3 (p=0.0366); other pairwise comparisons were not significant. For p53, the EV × grade interaction was not significant (exchangeable-correlation GEE Wald $\chi^2(3)=5.14$; p=0.162), with concordant working-independence (p=0.122) and bias-reduced covariance (p=0.264) analyses (Supplementary Table 2).

Categorical biomarker analyses. Global STING tertiles were 143.76 and 183.39 H-score units. EV tumors were more frequently in the high tertile (56.8% vs 20.5%) and less frequently in the low tertile (13.6% vs 44.9%); the cluster-robust ordinal OR was 5.12 (95% CI 2.60–10.09; $p=2.41\times10^{-6}$) and remained 5.66 after grade adjustment (2.82–11.37; $p=1.11\times10^{-6}$). For p53, tertile cutoffs were 23.09 and 92.97, with a cluster-robust OR of 3.54 (1.98–6.32; $p=1.97\times10^{-5}$) and grade-adjusted OR of 3.60 (1.97–6.56; $p=2.98\times10^{-5}$) (Supplementary Table 3). The separate <15/15–150/>150 p53 classification remained exploratory; strong expression was observed in 27% of EV versus 7% of non-EV tumors (FDR-adjusted p=0.006) (Supplementary Table 5).

STING–p53 association. The positive STING–p53 association persisted in patient-clustered GEE after adjustment for histological grade (β=0.258, 95% CI 0.136–0.380; $p=3.20\times10^{-5}$) and after additional adjustment for EV status (β=0.192, 0.074–0.310; p=0.00145).

Additional sensitivity analyses. Restricting analyses to tumors with at least two valid cores preserved the higher STING (median 194.0 vs 147.3; Cliff's δ=0.56; FDR-adjusted $p=3.66\times10^{-6}$) and p53 expression (103.3 vs 34.8; δ=0.44; p=0.00036), whereas BCL2 remained non-significant (2.46 vs 4.46; δ=−0.22; p=0.055). Arithmetic-mean and detection-weighted across-core aggregation produced the same directional conclusions. The original unclustered tumor-level Mann–Whitney and van Elteren analyses were retained as sensitivity analyses and were concordant for STING and p53 (Supplementary Table 5).
